# Diagnostic challenge of a cystic solid pseudopapillary tumor in pancreas: A case report

**DOI:** 10.1002/ccr3.4050

**Published:** 2021-03-20

**Authors:** Ebrahim Makhoul, Zeina Alabbas, Ali Adra, Alexey Youssef, Emad Ayoub, Rana Issa

**Affiliations:** ^1^ Faculty of Medicine Tishreen University Lattakia Syrian Arab Republic; ^2^ Al‐Mouwasat Hospital Damascus Syrian Arab Republic; ^3^ Oxford University Oxford UK

## Abstract

A solid pseudopapillary tumor should be included in the differential diagnosis of every pancreatic cystic lesion. A constellation of microscopic morphology and immunohistochemistry, in addition to the clinical history, aids in reaching the correct diagnosis.

## Graphical abstract

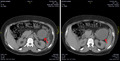

## 1 INTRODUCTION

Solid Pseudopapillary tumors of the pancreas (SPTPs) are rare tumors with nonspecific presentation which makes them a difficult diagnostic challenge. The morphologic features of the cells were similar to the cells seen in neuroendocrine tumors. Immunohistochemistry cleared up the doubts and made the diagnosis of SPTP the definitive diagnosis.

Solid Pseudopapillary tumors of the pancreas (SPTPs) are rare neoplasms that occur most commonly in females in their second or third decade and account for about 0.17%‐2.7% of all pancreatic tumors.^1^, ^2^

Abdominal pain is the most frequent clinical manifestation of SPTPs, and in the rest of the cases, there are no specific symptoms, wherein the diagnosis is made incidentally during routine examination.^3^

SPTPs are well defined neoplasms with solid and variably cystic areas. Occasionally, the extensive cystic tumor degeneration along with the bland morphology of SPTPs cells make it difficult to be differentiated from the other pancreatic tumors, especially neuroendocrine tumors; therefore, immunohistochemistry (IHC) plays a crucial role in making the accurate diagnosis. Here, we present a difficult‐to‐diagnose SPTP that manifested as a solitary pancreatic cyst.

## 2 CASE REPORT

A 36‐year‐old nonalcoholic female patient with a history of smoking for 15 years, presented with abdominal pain radiating to her back. The pain was not relieved by NSAIDS. The patient mentioned that she had experienced many episodes of nonbilious vomiting, nausea, and intermittent nonbloody diarrhea.

**FIGURE 1:**
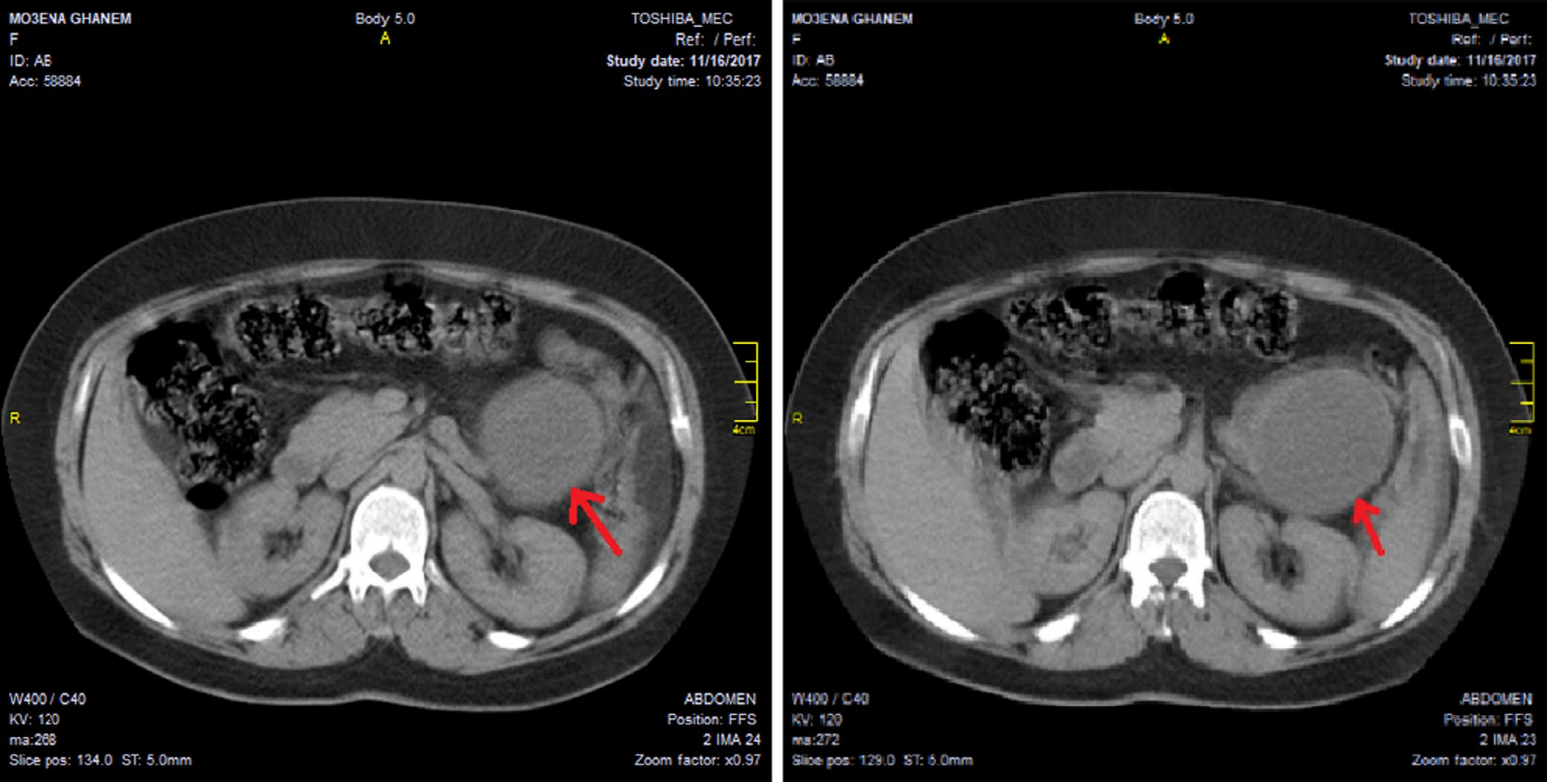
Noncontrast CT scan revealed unilocular cystic lesion in the tail of the pancreas attached to the spleen

On physical examination, a mass was palpated in the epigastric region. Laboratory tests were normal except for a mild anemia (Hemoglobin = 11 g/dL). The radiological findings on ultrasound (US) and noncontrast computed tomography (CT) revealed a unilocuar cystic lesion in the tail of the pancreas attached to the spleen (Figure 1).

The patient underwent distal pancreatectomy with splenectomy, and three regional lymph nodes were extracted. The samples were sent to the department of pathology.

**FIGURE 2:**
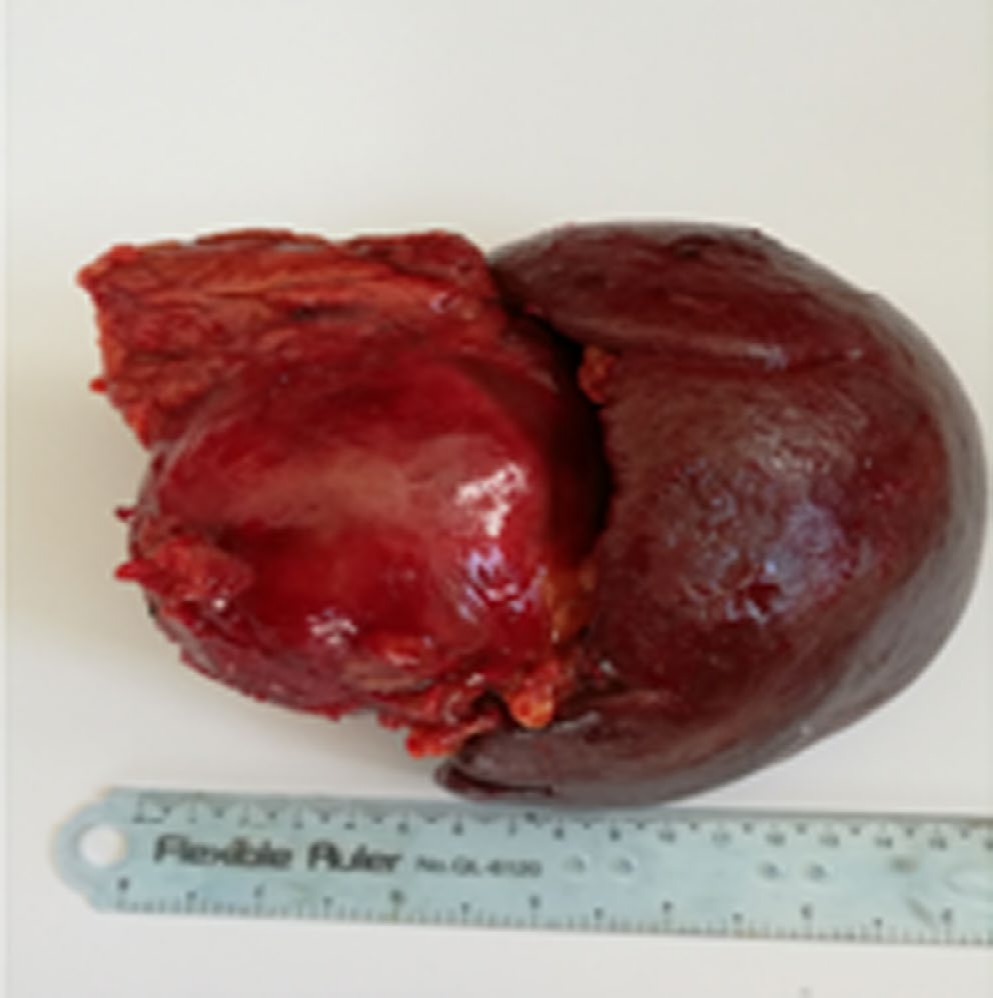
A Cystic mass in the distal part of the pancreas adherent to spleen

Grossly, the specimen was composed of distal part of the pancreas adherent to the spleen, where a cystic mass measuring about 8 cm in diameter was found, the rest of pancreas tissue measured 4 × 6 cm. The spleen measured 6 × 10 × 15 cm (Figure 2). The pathologist's first impression was a pancreatic pseudocyst, but other cystic neoplasms of pancreas could not be excluded. Later, the diagnosis of a pseudocyst was ruled out, as the patient had no history of pancreatitis or abdominal trauma.

**FIGURE 3:**
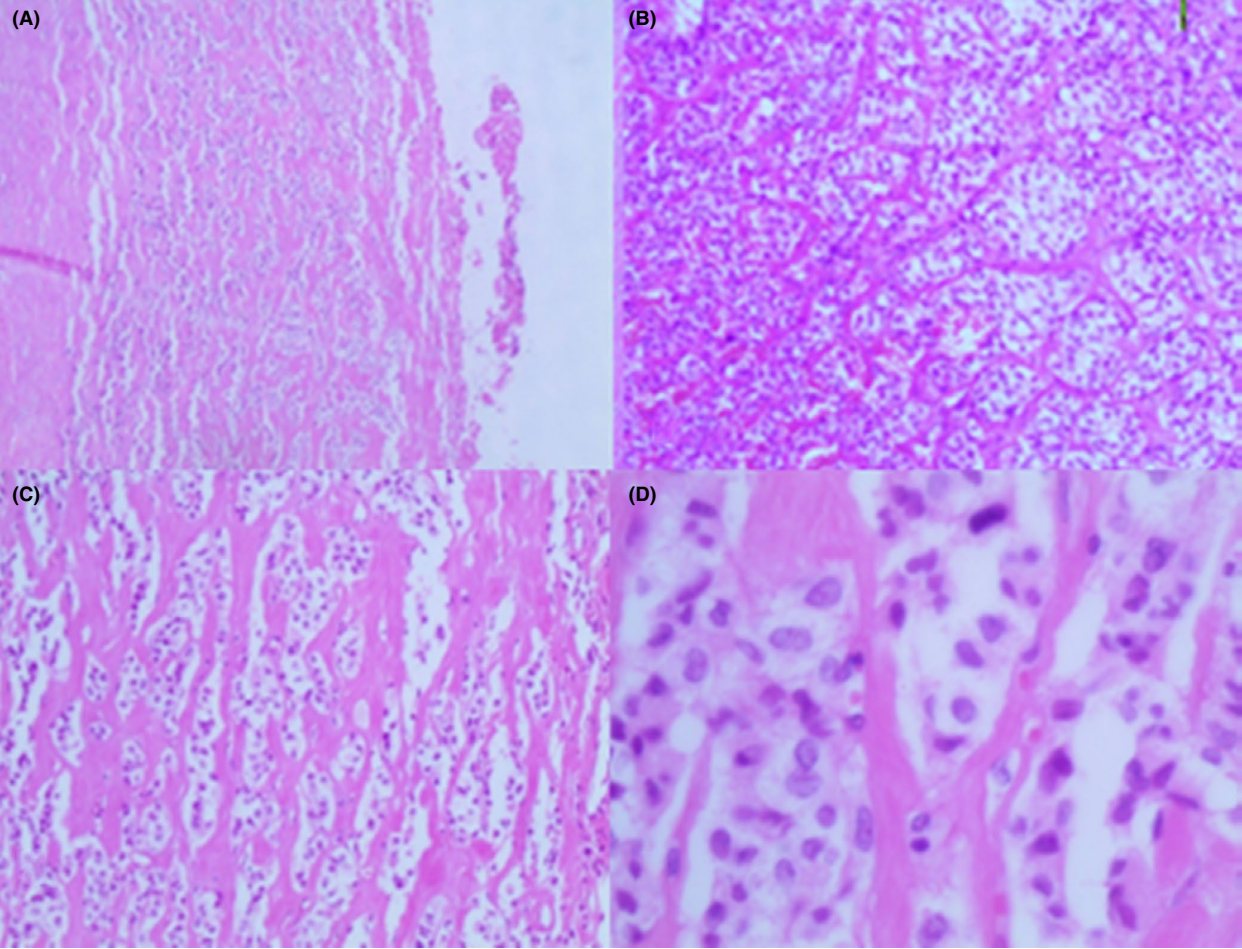
Microscopic findings of SPT, A, Nests of tumor cells embedded in fibrous capsule. B, C, and D the nests were composed of round monomorphic neoplastic cells. The cytoplasm was clear to granulate and the nuclei were uniform. (Haematoxylineosin, original magnification x40 [A] ×100 [B] ×200 [C] ×400 [D])

Microscopic examination of the H&E stained sections of the cyst wall revealed nests of neoplastic histiocyte - like cells embedded in a fibrovascular stroma. The cytoplasm was clear to granulate and the nuclei were uniform, round to oval with finely and evenly distributed chromatin (Figure 3). No mitotic figures or vascular invasion were identified. The spleen, all lymph nodes, and the surgical margins were tumor free.

**FIGURE 4:**
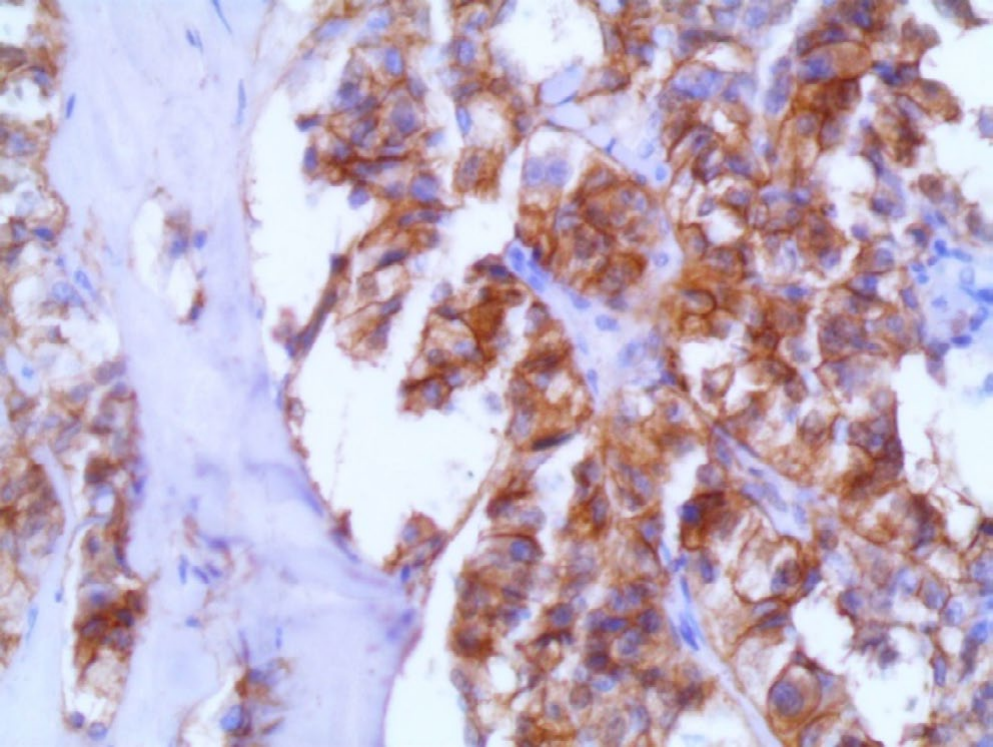
Tumor cells show positivity for CD56 (×400)

**FIGURE 5:**
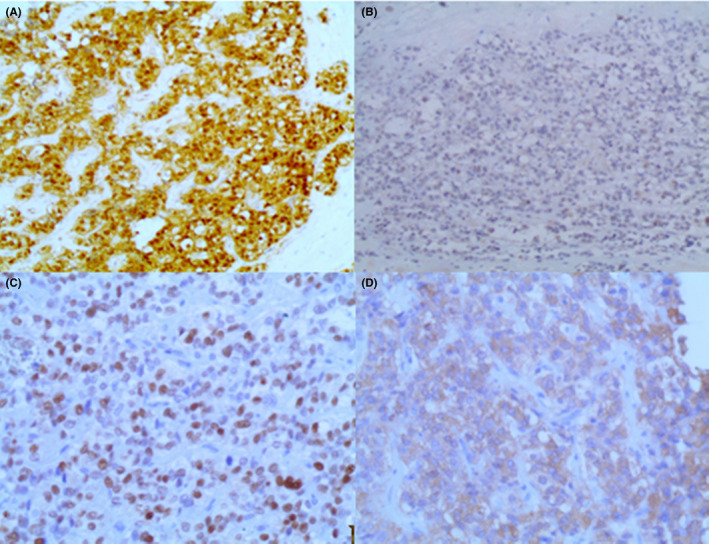
A, Positivity of β‐catenin in the tumor cells B, Ecadherin is diffusely negative in the tumor cells C, positivity of Cyclin D1 in the tumor cells. D, Tumor cells express weak positivity for NSE (Immunohistochemistry, orginal magnification ×100 [A, B] ×200 [C, D])

The IHC stains revealed positivity of the tumor cells for CD56 and cyclin D1 (Figures 4, 5). In addition, neuron‐specific enolase (NSE) was weakly positive, whereas CK7, CK20, pan cytokeratin (panCK), chromogranin A, synaptophisin, CD68, S100, vimentin, epithelial membrane antigen (EMA), and smooth muscle actin (SMA) were all negative.

Based on H&E stain and IHC results, the diagnosis was limited to SPT and NET of pancreas. However, a well‐differentiated endocrine tumor (G1‐NET) with almost total cystic degeneration was favored.

One year later, β‐catenin and E‐Cadherin were added to the IHC panel in our laboratory. The stains were then performed, and the tumor cells showed a negativity for E‐cadherin and expressed nuclear positivity for β‐catenin (Figure 5); consequently, the diagnosis of solid pseudopapillary tumor of pancreas was our final diagnosis.

## 3 DISCUSSION

Solid pseudopapillary tumors are rare low‐grade malignant neoplasms of uncertain cellular differentiation, commonly located in the tail of pancreas.^4^ Necrosis and cystic degeneration are common features in these tumors.^5^, ^6^

Clinically, SPTPs are usually nonsymptomatic and are discovered incidentally as abdominal masses during physical examinations.^3^ Laboratory tests are normal most of time. In our patient, epigastric pain with an abdominal mass was the main clinical presentation of the SPT.

Usually, the ultrasound and CT of SPTPs reveal well‐defined solid masses with cystic components.^7^ In our case, there was a solitary cyst in the tail of the pancreas.

Typically, the gross inspection of SPTPs shows well‐defined solid masses with variable areas of cystic degeneration.^8^ Microscopically, the tumors consist of solid nests of poorly cohesive uniform polygonal cells arranged in a solid pattern with frequent pseudopapillary structures resulting from the separation of the vessels with the attached tumor cells.^8^ In our case, the specimen contained a unilocular cyst without a solid component. In the H&E stained sections, there were nests of round monomorphic cells in the cyst wall, without significant cellular atypia. A large number of studies have been conducted to elucidate the histogenesis of these tumors, but the results were conflicted. Acinar, centroacinar, neural crest, and neuroendocrine origins were proposed by different investigation groups, but none of them has been proved. Furthermore, the highly variable immunohistochemical profiles of these tumors do not allow identifying precise cellular lineage.^9^ Recently, a gain of a function mutation in the gene encoding β‐catenin was reported in more than 90% of SPTPs.^8^ Moreover, there was a loss of E‐cadherin in all cases of SPTPs, and an overexpression of SOX11 protein, a member of SOX protein family modulators of Wnt/β‐catenin signaling pathway, in 82% of the cases, therefore, β‐catenin, E‐cadherin, and SOX11 were recommended as the most useful markers that may help to differentiate between SPTP and NET.^8^, ^9^, ^10^, ^11^, ^12^

In our case, the tumor cells showed positivity for CD56, NSE, and cyclin D1, and negativity for the epithelial and histocytic markers. It was difficult to distinguish between SPTP and low‐grade NET because of the microscopic and immunohistochemical overlaps. One year later, E‐cadherin and β‐catenin, but not SOX11, became available in our laboratory and performed to make the final diagnosis. The tumor cells showed negativity for E‐cadherin and positivity for β‐catenin, so we redeemed our diagnosis from NET to SPTP.

Surgery is the gold standard treatment with curative results if the lesion is completely resected.^4^ After about eighteen months of surgery, our patient showed neither signs of tumor recurrence nor endocrine and exocrine insufficiency of the pancreas.

## 4 CONCLUSION

Solid pseudopapillary tumors should be included in the differential diagnosis of every pancreatic cyst. A wide immunohistochemical panel, particularly β‐catenin and E‐cadherin, is needed to differentiate solid pseudopapillary tumors from other pancreatic neoplastic lesions, especially from neuroendocrine tumors.

## CONFLICT OF INTEREST

None declared.

## AUTHOR CONTRIBUTIONS

Ebrahim Makhoul and Zeina Alabbas: drafted the article and collected the patient's data. Ali Adra and Alexey Youssef: participated in collecting the data and drafting the article. Emad Ayoub: performed the surgical biopsy and participated in revising the article. Rana Issa: The guarantor and supervisor, critically revised the article, performed the pathologic examination, and approved the final manuscript.

## ETHICAL APPROVAL

Informed consent was obtained from the patient regarding the report of her clinical scenario data in an anonymous way.

## Data Availability

The data that support the findings of this study are available from the corresponding author upon reasonable request.
